# Columbia Open Health Data for COVID-19 Research: Database Analysis

**DOI:** 10.2196/31122

**Published:** 2021-09-30

**Authors:** Junghwan Lee, Jae Hyun Kim, Cong Liu, George Hripcsak, Karthik Natarajan, Casey Ta, Chunhua Weng

**Affiliations:** 1 Columbia University New York, NY United States

**Keywords:** COVID-19, open data, electronic health record, data science, research, data, access, database, symptom, cohort, prevalence

## Abstract

**Background:**

COVID-19 has threatened the health of tens of millions of people all over the world. Massive research efforts have been made in response to the COVID-19 pandemic. Utilization of clinical data can accelerate these research efforts to combat the pandemic since important characteristics of the patients are often found by examining the clinical data. Publicly accessible clinical data on COVID-19, however, remain limited despite the immediate need.

**Objective:**

To provide shareable clinical data to catalyze COVID-19 research, we present Columbia Open Health Data for COVID-19 Research (COHD-COVID), a publicly accessible database providing clinical concept prevalence, clinical concept co-occurrence, and clinical symptom prevalence for hospitalized patients with COVID-19. COHD-COVID also provides data on hospitalized patients with influenza and general hospitalized patients as comparator cohorts.

**Methods:**

The data used in COHD-COVID were obtained from NewYork-Presbyterian/Columbia University Irving Medical Center’s electronic health records database. Condition, drug, and procedure concepts were obtained from the visits of identified patients from the cohorts. Rare concepts were excluded, and the true concept counts were perturbed using Poisson randomization to protect patient privacy. Concept prevalence, concept prevalence ratio, concept co-occurrence, and symptom prevalence were calculated using the obtained concepts.

**Results:**

Concept prevalence and concept prevalence ratio analyses showed the clinical characteristics of the COVID-19 cohorts, confirming the well-known characteristics of COVID-19 (eg, acute lower respiratory tract infection and cough). The concepts related to the well-known characteristics of COVID-19 recorded high prevalence and high prevalence ratio in the COVID-19 cohort compared to the hospitalized influenza cohort and general hospitalized cohort. Concept co-occurrence analyses showed potential associations between specific concepts. In case of acute lower respiratory tract infection in the COVID-19 cohort, a high co-occurrence ratio was obtained with COVID-19–related concepts and commonly used drugs (eg, disease due to coronavirus and acetaminophen). Symptom prevalence analysis indicated symptom-level characteristics of the cohorts and confirmed that well-known symptoms of COVID-19 (eg, fever, cough, and dyspnea) showed higher prevalence than the hospitalized influenza cohort and the general hospitalized cohort.

**Conclusions:**

We present COHD-COVID, a publicly accessible database providing useful clinical data for hospitalized patients with COVID-19, hospitalized patients with influenza, and general hospitalized patients. We expect COHD-COVID to provide researchers and clinicians quantitative measures of COVID-19–related clinical features to better understand and combat the pandemic.

## Introduction

COVID-19 has threatened the health of tens of millions of people all over the world. The global pandemic caused by COVID-19 has sparked massive research efforts in the fight against the novel disease, including characterizing the disease and clinical progression, identifying risk factors for hospitalization, and finding drugs that can be repurposed to lessen disease severity [1-3]. Utilization of clinical data from different institutions, hospitals, and nations can accelerate these research efforts since important characteristics of the patients are often found by examining the shared clinical data. Although many studies sharing epidemiological data [4,5], public health [6], and social measures [7] for COVID-19 research have been conducted, publicly accessible clinical data on COVID-19 remain limited despite the immediate need [8], mainly owing to the potential risk to patient privacy that can still exist even after deidentification of the data [9].

Recognizing the need for publicly accessible electronic health record (EHR)–derived data in a broad range of clinical and translational research, we previously developed Columbia Open Health Data (COHD). COHD provides open and easy access to prevalence and co-occurrence statistics on conditions, drugs, procedures, and demographics derived from structured EHR data from NewYork-Presbyterian/Columbia University Irving Medical Center (NYP/CUIMC) [10], which serves the large and diverse population of NYC and its surrounding areas. Since its deployment, COHD has accelerated biomedical research by providing 2 informative resources, prevalence and co-occurrence statistics, and their derived association metrics [10-12].

NYC was one of the first epicenters of COVID-19 in the United States with the first confirmed case on February 29, 2020 [13]. As one of the largest academic medical centers in NYC, NYP/CUIMC has admitted more than 4000 patients as of September 1, 2020. We aim to provide shareable clinical data to catalyze future COVID-19 research by presenting Columbia Open Health Data for COVID-19 Research (COHD-COVID), a publicly accessible database providing clinical concept prevalence, the clinical concept prevalence ratio between cohorts, clinical concept co-occurrence, and clinical symptom prevalence for a cohort of hospitalized patients with COVID-19 and comparator cohorts (a cohort of hospitalized patients with influenza and a general hospitalized patient cohort) derived from NYP/CUIMC’s electronic health records. In addition to providing publicly accessible data files via the Figshare data repository, we also developed the COHD-COVID web application programming interface (API) for easy access and better usability.

## Methods

### Methods Overview

We used the term “concept” to refer to clinical entities and events such as conditions (ie, diagnosis), drugs, and procedures. The concepts and their names are defined by the Observational Medical Outcomes Partnership (OMOP) Common Data Model (CDM). When concepts are referenced in this paper, the name of the concept is styled in italics (eg, *Disorder of respiratory system*) to distinguish the formalized concepts from regular text. We also styled entities in the OMOP CDM (eg, *person_id* column in *condition_occurrence* table) in italics.

**Figure 1:**
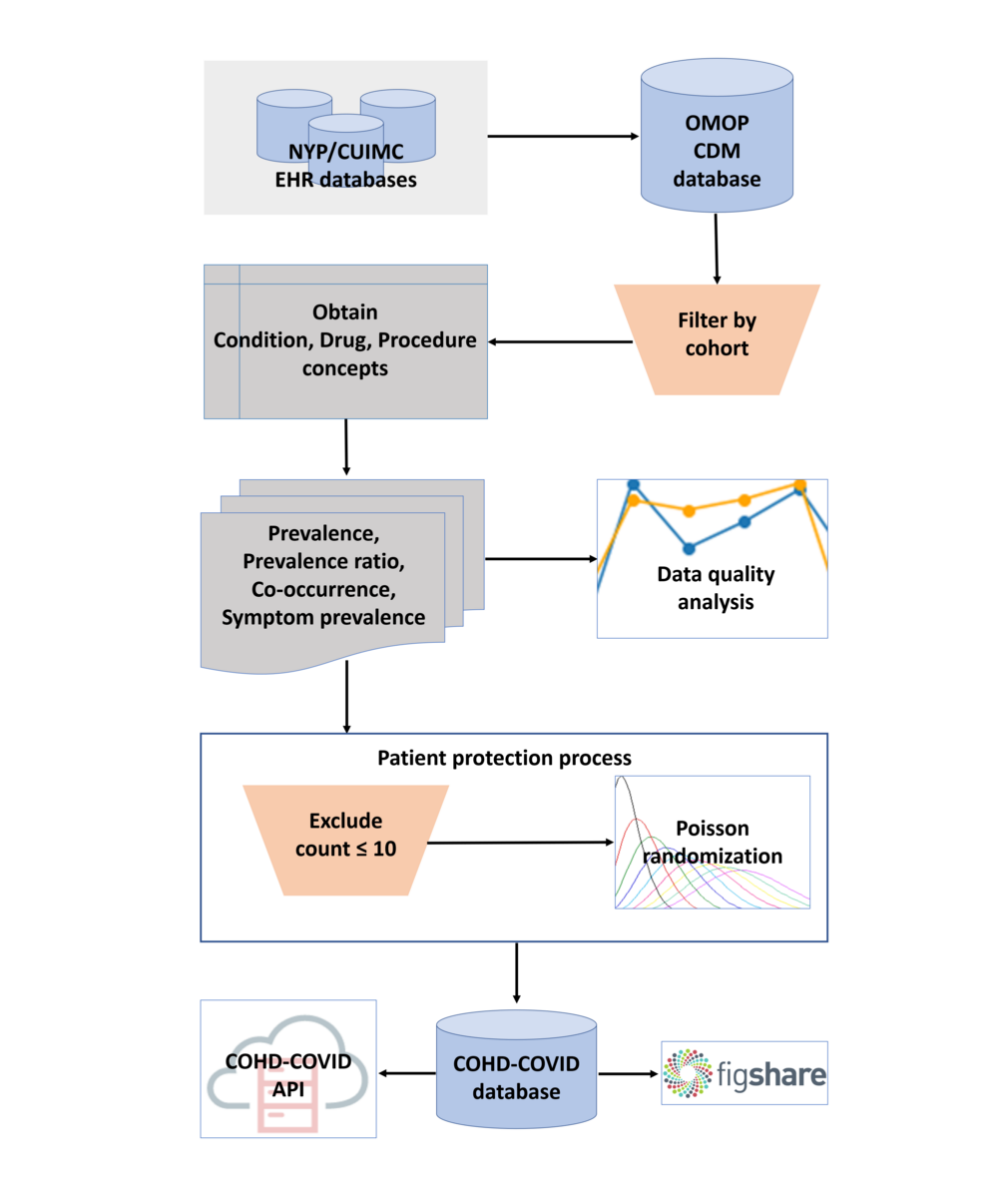
Overall workflow of Columbia Open Health Data for COVID-19 Research (COHD-COVID). API: application programming interface; NYP/CUIMC: NewYork-Presbyterian/Columbia University Irving Medical Center; OMOP CDM: Observational Medical Outcomes Partnership Common Data Model.

Figure 1 depicts the overall workflow to create COHD-COVID. Columbia’s clinical data warehouse was converted to the OMOP CDM. We first filtered the EHR data in accordance with the cohort definitions, and then EHR for each patient’s inpatient visits were identified. Condition, drug, and procedure concepts were obtained from the identified visits. Concept prevalence, concept prevalence ratio, concept co-occurrence, and symptom prevalence analyses followed using the obtained concepts. To protect patient privacy, we excluded rare concepts observed in 10 or fewer visits and perturbed the true counts using Poisson randomization. Perturbation of the true counts and exclusion of rare concepts reduce the uniqueness of individual in the data, which can minimize the risk of reidentification [9]. Perturbed counts generated by the Poisson randomization process do not show a significant difference from the true counts [10]. The resulting data were stored in a MySQL database and made publicly available via the COHD-COVID web API [14]. All analyses were conducted using Python 3.5.2. This study received institutional review board approval with a waiver for informed consent.

### Data Source

We utilized EHR data from the NYP/CUIMC’s clinical data warehouse, where inpatient and outpatient data dating back to 1985 are stored. EHR data were collected during routine clinical care. Patients were notified of potential uses of their data for research at the point of care and data collection. NYP/CUIMC has converted its clinical data warehouse to OMOP CDM on a regular basis. Since NYP/CUIMC covers NYC and the surrounding area, which has a diverse population of 8.2 million people and was an early COVID-19 epicenter, the EHR data from NYP/CUIMC can provide a diverse and large sample of patients with COVID-19.

Three different patient cohorts were used in this study. The COVID-19 cohort was defined as hospitalized patients aged ≥18 years with a COVID-19–related condition diagnosis or a confirmed positive COVID-19 test result during their hospitalization period or within the prior 21 days. Patients identified with the COVID-19 cohort definition from March 1, 2020, to September 1, 2020, were included in the COVID-19 cohort. The influenza cohort was similarly defined as patients aged ≥18 years who had at least 1 occurrence of influenza conditions or precoordinated positive measurements or positive influenza test results during their hospitalization period or within the prior 21 days. The general cohort was defined as all hospitalized patients aged ≥18 years. Patient visits from calendar years 2014 to 2019 were included for the influenza and general cohorts. All cohorts were divided into subcohorts stratified by sex (male vs female) and age (adults aged 18-64 years vs seniors aged >65 years) for further investigation. The COVID-19 and influenza cohort definitions were adapted from the cohort definitions created by the Observational Health Data Science and Informatics’ (OHDSI’s) international network study for COVID-19 [3]. Summary statistics of the cohorts are provided in Table 1.

Patients belonging to the cohorts based on the 3 cohort definitions above were identified using the unique *person_id* from the *person* table in the OMOP CDM. Condition, drug, and procedure concepts observed in these patients during inpatient visits were extracted from the *condition_occurrence*, *drug_exposure*, and *procedure_occurrence* tables in the OMOP CDM, respectively. Inpatient visits of patients and the concepts in these visits were identified using *person_id* along with *visit_occurrence_id* from the *visit_occurrence* table. We used visit-based counts instead of patient-based counts in the following analyses for robust comparison between cohorts that had different observation windows. For example, if we use patient-based counts, the patients included in cohorts with longer observation windows are likely to be observed with more concepts than the patients in cohorts with shorter observation windows, which could inject bias into the metrics used in the analyses. Thus, we used visit-based counts to mitigate the effect of different observation window lengths to reduce the bias.

**Table 1:** Basic statistics of 3 cohorts in the Columbia Open Health Data for COVID-19 Research database. The statistics summarized here are based on the data from NewYork-Presbyterian/Columbia University Irving Medical Center as of September 1, 2020.

	COVID-19 cohort	Influenza cohort	General cohort
Time range	March 1, 2020, to August 31, 2020	January 1, 2014, to December 12, 2019	January 1, 2014, to December 12, 2019
Patients, n	4127	3261	175,930
Total inpatient visits, n	4846	3860	314,680
Male patients; inpatient visits, n (%)	2103 (51.0); 2518 (52.0)	1454 (44.6); 1732 (44.9)	67,662 (38.5); 128,908 (41.0)
Female patients; inpatient visits, n (%)	2024 (49.0); 2328 (48.0)	1807 (55.4); 2128 (55.1)	108,268 (61.5); 185,772 (59.0)
Adult patients (aged 18-64 years); inpatient visits, n (%)	2147 (52.0); 2511 (51.8)	1315 (40.3); 1553 (40.2)	104,020 (59.1); 173,843 (55.2)
Senior patients (>65 years); inpatient visits, n (%)	1980 (48.0); 2335 (48.2)	1946 (59.7); 2307 (59.8)	71,910 (40.9); 140,837 (44.8)

### Concept Prevalence and Concept Prevalence Ratio Analysis

We calculated the concept prevalence in each cohort as detected from the EHR. The concept prevalence is defined as in equation **(1)**.



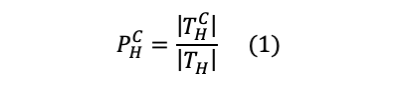



where 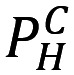 is the prevalence of concept *C* in cohort *H*, 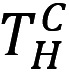 is the set of unique inpatient visits of patients in cohort *H* observed with concept *C*, and *T_H_* is the set of unique inpatient visits of patients in cohort *H.* We also calculated hierarchical concept prevalence by defining 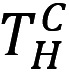 as the set of unique visits of patients observed with concept *C* or any of concept *C*’s descendant concepts as defined in the *concept_ancestor* table in the OMOP CDM. For example, the hierarchical count for *Ibuprofen* (OMOP concept ID 1177480) not only includes entries where the specific concept *Ibuprofen* was used, but also includes entries using other descendant concepts, such as *Ibuprofen 600 MG Oral Tablet* (OMOP concept ID 19019073). Taking hierarchical relationships into account mitigates some of the issues with coding variations across time and practices as different concepts with minor semantic differences can be aggregated into higher-level concepts.

The concept prevalence ratio indicates how frequently concept *C* occurs in cohort *A* relative to cohort *B*. The natural logarithm of the concept prevalence ratio is defined as in equation **(2)**.



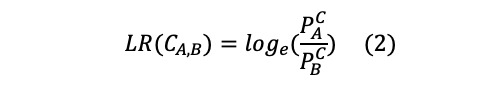



where *LR*(*C_A,B_*) is the log ratio of the prevalence of concept *C* for cohort *A* to cohort *B*, 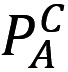 is prevalence of concept *C* in cohort *A*, and 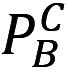 is prevalence of concept *C* in cohort *B*. Hierarchical concept prevalence ratio can be calculated by using hierarchical concept prevalence 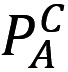 and 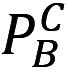.

### Concept Co-occurrence Analysis

Concept co-occurrence represents how frequently a specific concept pair appears in a cohort. We defined concept co-occurrence prevalence as in equation **(3)**.



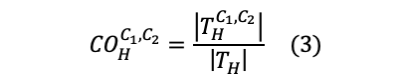



where 
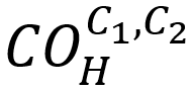
 is the co-occurrence prevalence of concepts *C_1_* and *C_2_* in cohort *H*, 
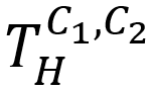
 is the set of unique visits of patients observed with concept *C_1_* and *C_2_* in cohort *H*, and *T_H_* is the set of unique visits of patients in the cohort *H*. We also calculated hierarchical concept co-occurrence using the hierarchy of concepts as described above.

### COVID-19 Symptom Prevalence Analysis

Since clinical symptoms often include multiple granular clinical concepts, a set of related concepts for a symptom is required to calculate the prevalence of the symptom. For example, dyspnea, which is one of the major symptoms of COVID-19, can be detected as standard concept *Dyspnea* or *Acute respiratory distress* in different patients. The 2 concepts do not have any hierarchical relationship and thus will not be aggregated by the hierarchical prevalence analyses. Thus, a concept set containing both *Dyspnea* and *Acute respiratory distress* is needed for accurate calculation of the prevalence of dyspnea. We defined symptom prevalence as in equation **(4)**.



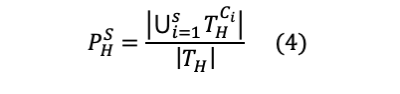



where 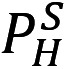 is prevalence of symptom *S* in cohort *H*, 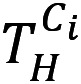 is the set of unique visits of patients observed with concept *C_i_*, *T_H_* is the set of unique visits of patients observed in the cohort *H*, *s* is the number of the unique concepts in the concept set for symptom *S*, and ∪ is the union operator. Hierarchy between concepts is not considered in the symptom prevalence analysis since a concept set of a symptom already reflects hierarchy for that symptom.

We used concept sets for 11 major symptoms of COVID-19 (*Cough*, *Chills*, *Abdominal pain*, *Diarrhea*, *Dyspnea*, *Fatigue*, *Fever*, *Myalgia*, *Nausea and vomiting*, *Tachypnea*, and *Throat pain*), which have been defined by OHDSI to calculate symptom prevalence. The concepts included in each symptom are available in Figshare [15].

### Data Quality Analysis

Assessing the quality of EHR data is critical for its effects on secondary analysis for research in the health care and medical domains [16]. We calculated the sum of nonrandomized counts of all concepts on a monthly basis for the COVID-19 cohort and on a yearly basis for the general and influenza cohorts. For each of the condition, drug, and procedure domains, we examined the total counts of concepts with the number of visits to detect any issues regarding data quality and temporal plausibility of the EHR data we used in the study [17]. We also calculated the annual mean (SD) values of concept prevalence and concept co-occurrence for the general and influenza cohorts to assess the temporal variance of each concept and concept co-occurrence pair.

## Results

### Results Overview

In this section, we show a sample of the results of analyses using the data from COHD-COVID. Since COHD-COVID contains massive amounts of data covering several thousand concepts, it is worth noting that only a small sample of the results is shown in this section. Users can obtain the results of interest in addition to the results shown in this section by using the COHD-COVID API [14] or by downloading the flat data files from Figshare [15]. The results of all analyses, concept definitions, and concepts included in each symptom are available as tab-delimited flat files in Figshare, except the concept prevalence ratio analyses, since they can be directly computed from concept prevalence data. COHD-COVID API [14] provides all results.

### Concept Prevalence Analysis

**Figure 2:**
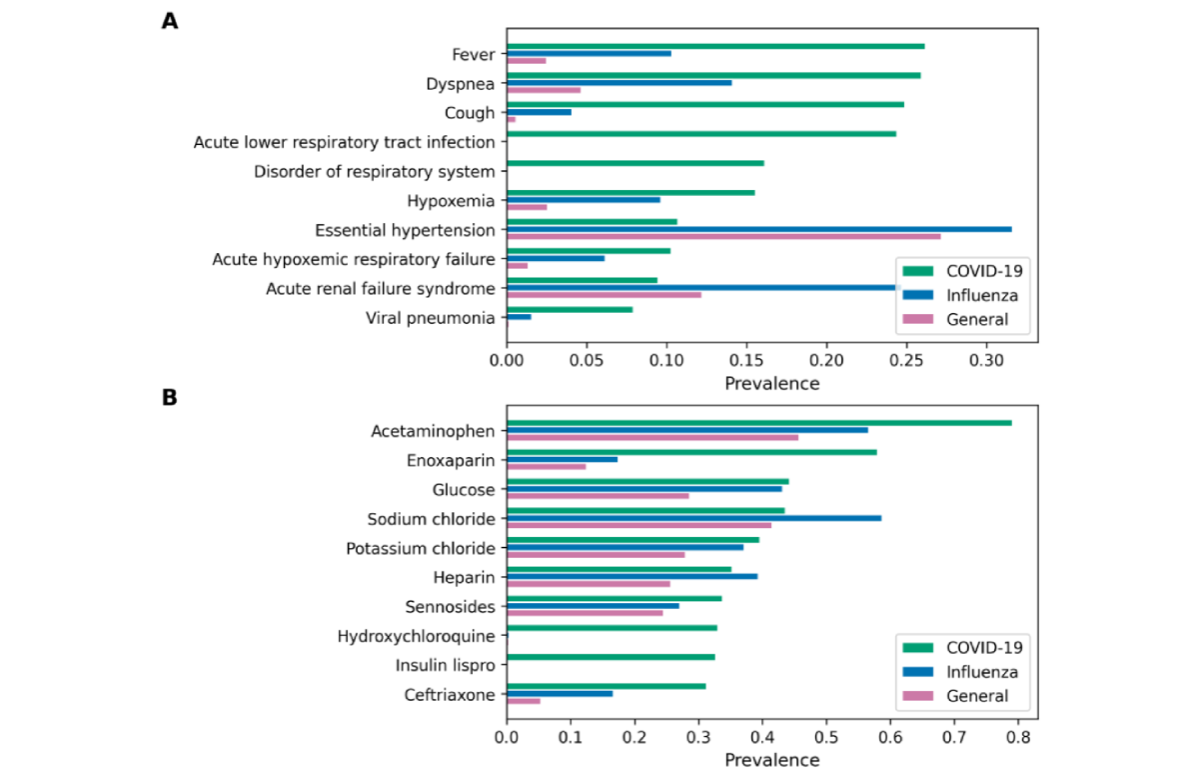
(A) Condition and (B) drug concept prevalence in the COVID-19 cohort, influenza cohort, and general cohort.

**Figure 3:**
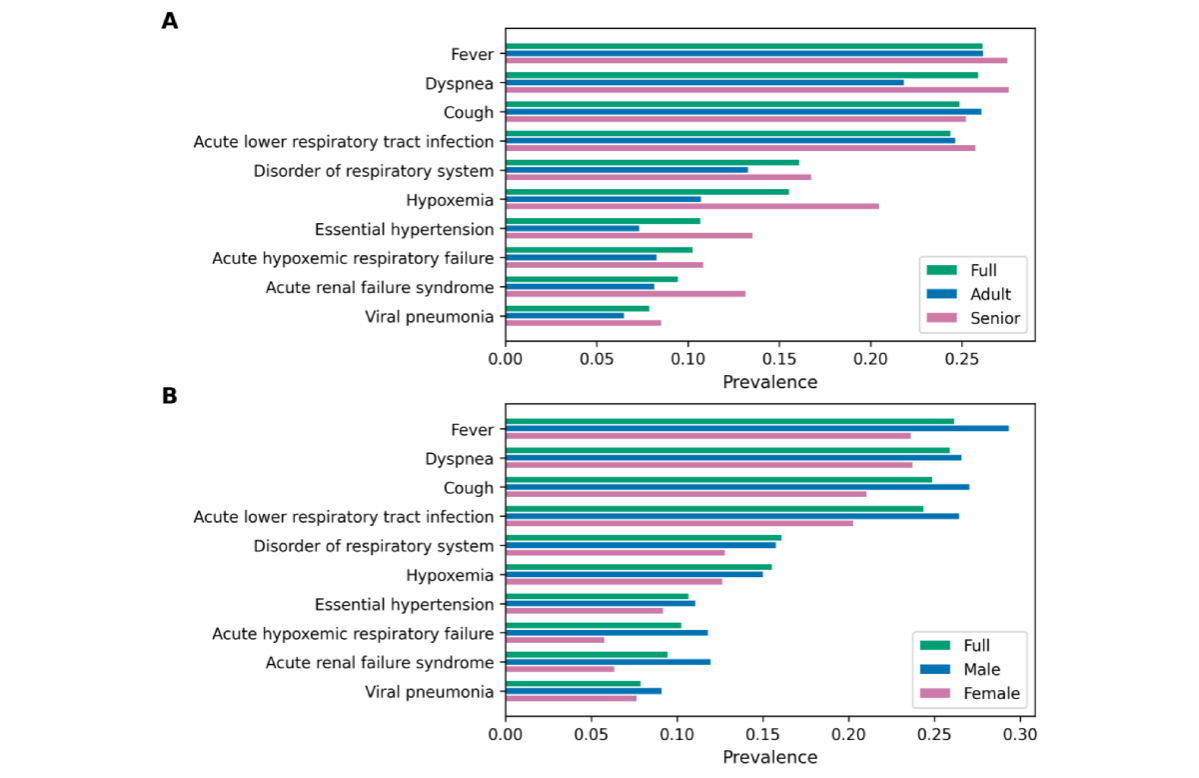
Condition concept prevalence in (A) age and (B) sex sub-cohorts of the COVID-19 cohort. The full COVID-19 cohort indicates original COVID-19 cohort without stratification.

**Figure 4:**
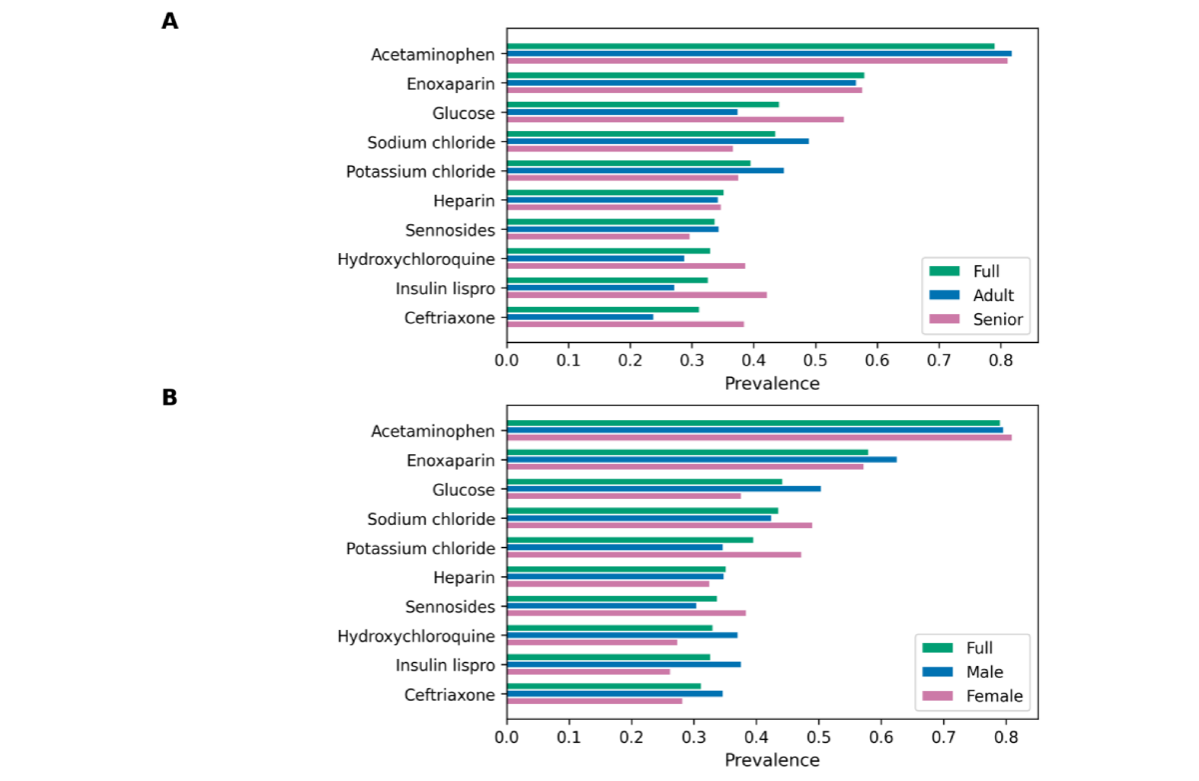
Drug concept prevalence in (A) age and (B) sex subcohorts of the COVID-19 cohort. The full COVID-19 cohort indicates original COVID-19 cohort without stratification.

Figure 2 shows the prevalence of 10 condition (Figure 2A) and drug concepts (Figure 2B) in the COVID-19 cohort, influenza cohort, and general cohort. We chose the 10 most prevalent condition and drug concepts in the COVID-19 cohort for this use case. For drug concepts, we chose the most prevalent drug ingredient concepts using hierarchical analysis to count multiple drugs that have different brand names, dosages, and formulations but are based on the same ingredients together. The condition concepts were chosen without hierarchical analysis to identify the top 10 specific conditions. *Fever* showed the highest prevalence (0.2619) among all condition concepts for the COVID-19 cohort, followed by *Cough* (0.2491) and *Dyspnea* (0.2594). *Acetaminophen* was the most prevalent (0.7912) drug ingredient used for patients with COVID-19, followed by *Enoxaparin* (0.5803) and *Glucose* (0.4424). Figures 3 and 4 show the prevalence of 10 condition and drug concepts, respectively, in the COVID-19 cohort stratified by age (Figures 3A and 4A) and gender (Figure 4A and 4B). The 10 condition and drug concepts were the top 10 most prevalent concepts in the full COVID-19 cohort without stratification.

### Concept Prevalence Ratio Analysis

Table 2 shows the top 10 condition concepts that showed the highest concept prevalence ratio for the COVID-19 cohort relative to the comparator cohorts (general cohort and influenza cohort). *Disease due to Coronavirus* and *Acute lower respiratory tract infection* showed the highest concept prevalence ratio for the COVID-19 cohort relative to the influenza cohort and the general cohort, respectively.

**Table 2:** Top 10 condition concepts that show the highest concept prevalence ratio for the COVID-19 cohort relative to the general and influenza cohorts.

Influenza cohort (prevalence ratio)	General cohort (prevalence ratio)
Disease due to Coronavirus (3.28)	Acute lower respiratory tract infection (7.30)
Disorientated (2.44)	Disorder of respiratory system (5.56)
Blood chemistry abnormal (2.25)	General finding of observation of patient (5.17)
Acute respiratory distress syndrome (2.16)	Outcome of delivery – finding (5.13)
Cerebral infarction (1.84)	Chest pain on breathing (5.09)
Cough (1.81)	Disease due to Coronavirus (4.95)
Viral pneumonia (1.61)	Patient status finding (4.60)
Acute respiratory distress (1.52)	Unplanned pregnancy (4.23)
Heart disease (1.32)	Acute respiratory distress syndrome (4.17)
Delivery normal (1.28)	Deliveries by cesarean (3.98)

### Concept Co-occurrence Analysis

Table 3 shows the top 10 most frequently co-occurring concepts with *Acute lower respiratory tract infection* in the COVID-19 cohort. *Acute lower respiratory tract infection* was chosen among the 10 most prevalent condition concepts in the COVID-19 cohort. We used nonhierarchical concept co-occurrence to obtain the result.

**Table 3:** The 10 concepts that most frequently co-occurred with Acute lower respiratory tract infection in the full COVID-19 cohort.

Concept name	Co-occurrence prevalence
Disease due to Coronavirus	0.2329
Radiologic examination, chest; single view	0.2214
Infectious agent detection by nucleic acid (DNA or RNA); severe acute respiratory syndrome coronavirus 2	0.2205
Acetaminophen 325 MG Oral Tablet	0.2037
0.4 ML Enoxaparin sodium 100 MG/ML Prefilled Syringe	0.1504
Hydroxychloroquine Sulfate 200 MG Oral Tablet	0.1246
50 ML Glucose 500 MG/ML Prefilled Syringe	0.1085
Ceftriaxone 1000 MG Injection	0.1073
Blood typing, serologic; ABO	0.1044
Glucose 0.4 MG/MG Oral Gel	0.0970

### COVID-19 Symptom Prevalence Analysis

**Figure 5:**
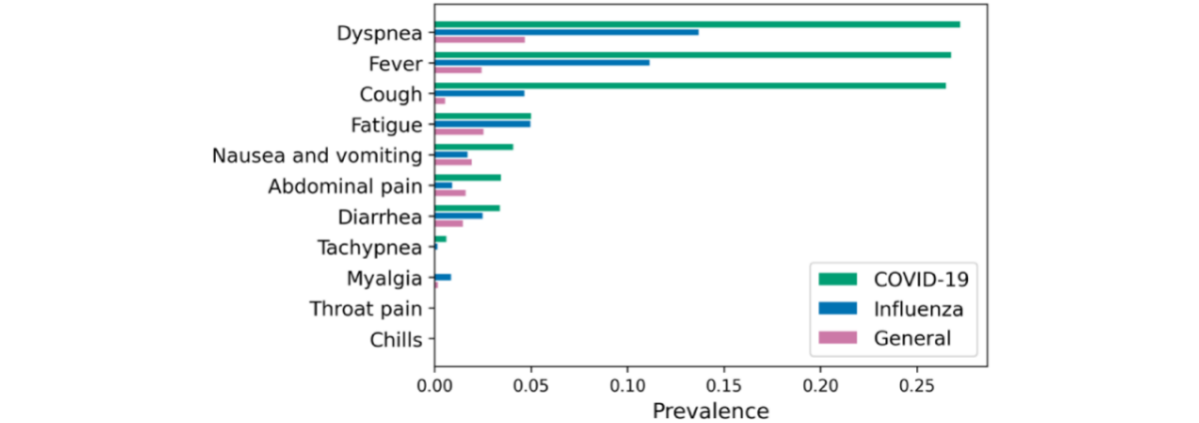
Symptom prevalence of 11 major symptoms in patients with COVID-19 for all 3 cohorts.

Figure 5 shows the prevalence of the 11 major COVID-19 symptoms for all 3 cohorts. In the COVID-19 cohort, *Dyspnea* showed the highest prevalence among the 11 symptoms followed by *Fever* and *Cough*.

### Data Quality Analysis

**Figure 6:**
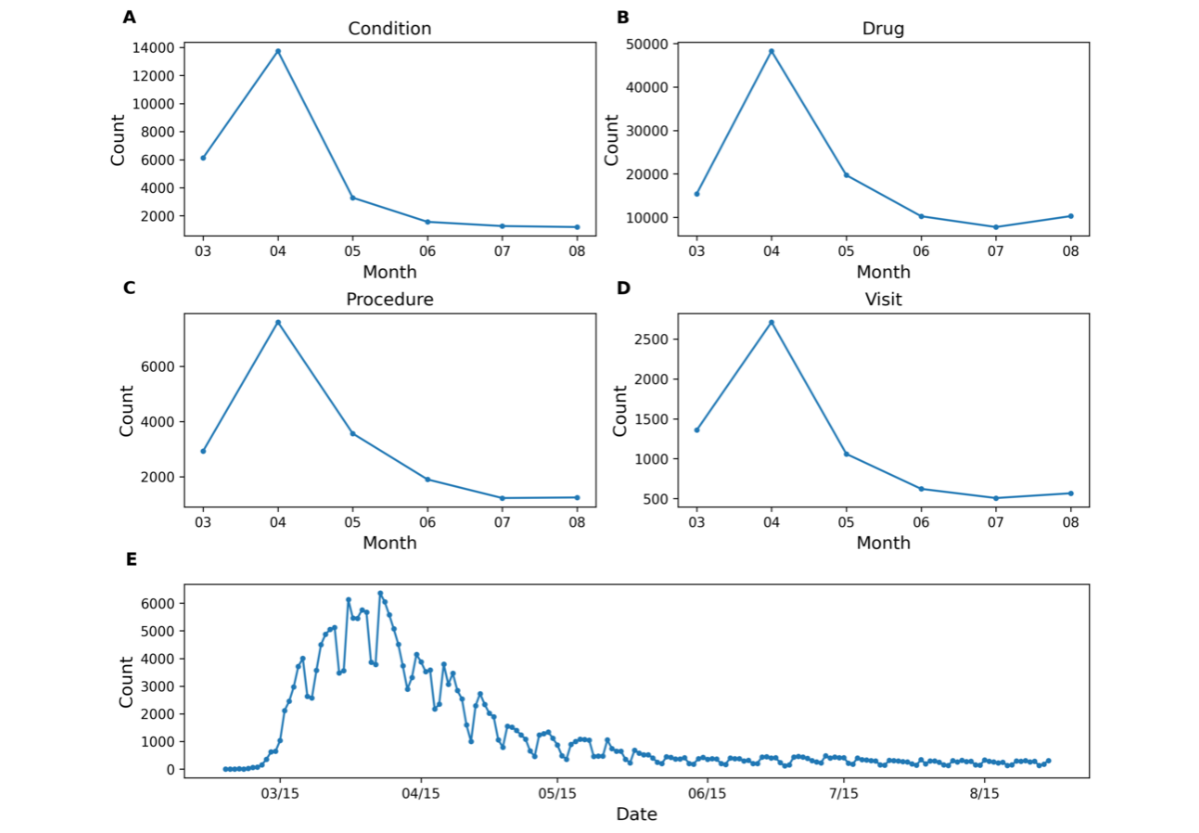
Total counts across all concepts in the (A) condition domain, (B) drug domain, (C) procedure domain, and (D) the total visits per month between March 2020 and August 2020 for the COVID-19 cohort. (E) Total COVID-19–positive cases per day in New York City from March 2020 to August 2020.

Figure 6 shows the total counts across all concepts in the condition domain (Figure 6A), drug domain (Figure 6B), procedure domain (Figure 6C), and the total visits per month between March 2020 and August 2020 (Figure 6D) for the COVID-19 cohort. The total counts of conditions, drugs, procedures, and total visits all show steep increases in March and April 2020, when the number of COVID-19 cases surged in NYC (Figure 6E) [18]. The total counts of all domains and the total visits decreased starting May 2020 as the number of patients with COVID-19 in NYC decreased.

**Figure 7:**
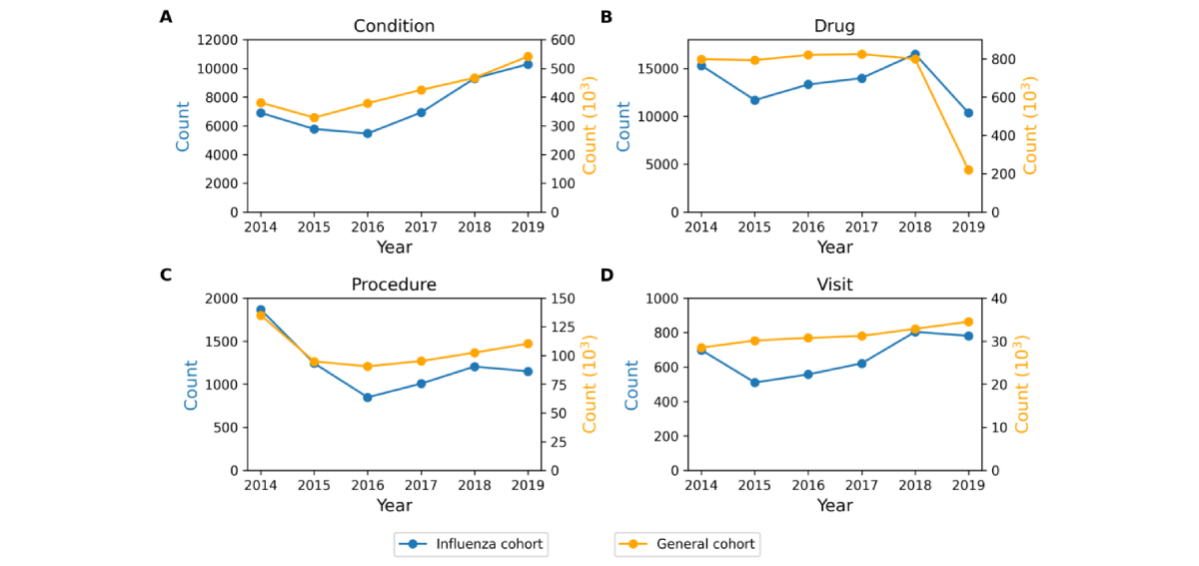
Total counts across all concepts in the (A) condition domain, (B) drug domain, (C) procedure domain, and (D) the total visits per year between 2014 and 2019 for the general and influenza cohort.

Figure 7 shows the total counts per year across all concepts in the condition domain (Figure 7A), drug domain (Figure 7B), procedure domain (Figure 7C), and the total visits per year between 2014 and 2019 (Figure 7D) for the general cohort and influenza cohort. The total counts of conditions, drugs, procedures, and total visits per year for the 2 cohorts show consistent trends.

The annual mean (SD) value of concept prevalence and concept co-occurrence for the general and influenza cohorts are available in Figshare [15] to assess the temporal variance of each concept and concept co-occurrence pair. The mean and standard deviation of annual concept prevalence and co-occurrence should only be compared to each other to assess the stability of the concept over the given time period of the data set.

A change in the EHR system can affect the quality and characteristics of EHR data collected for secondary research. NYP/CUIMC changed its EHR system as of February 1, 2020, from Allscripts to Epic, which might affect the COVID-19 cohort data and its characteristics as opposed to the influenza and general cohorts, which were collected prior to the EHR change. To detect the impact of the change, we performed a *t* test for all concepts reported in the COVID-19 cohort between the counts from a pre-Epic period (January 1, 2020, to January 31, 2020) and from a post-Epic period (February 1, 2020, to February 29, 2020). The post-Epic period was chosen to minimize the inclusion of dates when COVID-19 would have impacted clinical practices in NYC. The counts of concepts were recorded on a daily basis. We considered that there would be no difference between the 2 periods if the counts for the concept from the 2 periods are the same (ie, all 0 counts for the 2 periods). Of all 1066 unique concepts reported in the COVID-19 cohort, 119 (11.2%) concepts showed a significant difference (*P*≤.01) between the pre- and post-Epic periods. The *P* values of the *t* tests for all individual concepts are available in Figshare [15] to allow users to factor in these data quality considerations for each concept.

## Discussion

### Principal Findings

In this study, we present a publicly accessible database providing clinical concept prevalence, clinical concept co-occurrence, and clinical symptom prevalence for hospitalized patients with COVID-19, hospitalized patients with influenza, and general hospitalized patients. We showed the utility of the data for future data-driven studies by exploring the results of the concept prevalence analysis, concept prevalence ratio analysis, concept co-occurrence analysis, and symptom prevalence analysis. Results from the analyses aligned with published findings and can be used to find novel hypotheses as described in the following discussion. The results of all analyses were provided as flat files in Figshare [15] and also easily accessible through web API [14].

Concept prevalence analysis on the 3 cohorts can be used to determine the clinical characteristics of the COVID-19 cohort. A comparison of the prevalence of these concepts between the COVID-19 cohort and the influenza or general cohorts provides contextual evidence regarding whether the concept is associated with COVID-19 or if it is common among hospitalized patients. Figure 2A shows that *Fever*, *Cough*, *Dyspnea*, and *Acute lower respiratory syndrome* were more highly prevalent in the COVID-19 cohort than in the 2 comparator cohorts. The concepts highly prevalent in the COVID-19 cohort were well-known symptoms of COVID-19, concurrent with existing studies. As shown in Figure 2B, *Hydroxychloriquine*, which was one of the drugs widely administered to patients with COVID-19 [19] during the early months of the pandemic, showed high prevalence in the COVID-19 cohort.

Concept prevalence analysis on the COVID-19 cohort and its subcohorts can be used to determine the clinical characteristics of the subcohorts and determine if these characters differ between the sexes or between adult and older patients. Figure 3A shows that the senior cohort had higher prevalences in all 10 condition concepts than the full and adult cohorts and also shows that *Essential hypertension* and *Hypoxemia* were particularly more prevalent in senior patients with COVID-19, which indicates that senior patients are more likely to have these apparent symptoms of COVID-19 than adult patients. Figure 4A shows that drug ingredients related to type 2 diabetes (eg, *Glucose* and *Insulin Lispro*) showed higher prevalence in senior patients than in adult patients, which indicates that type 2 diabetes is a more common comorbidity among senior patients with COVID-19.

Concept prevalence ratio analysis can be used to unveil how often specific concepts appeared in the COVID-19 cohort compared to the comparator cohorts. Table 2 shows that common and general symptoms of COVID-19 (eg, *Chest pain on breathing*, *Cough*, and *Disease due to coronavirus*) showed a high prevalence ratio in the COVID-19 cohort relative to the general cohort. In contrast, more severe symptoms of COVID-19 (eg, *Disoriented*, *Acute respiratory distress syndrome*, *Viral pneumonia*, and *Blood chemistry abnormal*) showed a higher prevalence ratio in the COVID-19 cohort than in the influenza cohort. Since both cohorts include hospitalized patients, these results may indicate that the aforementioned conditions are more strongly associated with the SARS-CoV-2 infection than with influenza. We also observed high prevalence ratios in concepts related to delivery in the COVID-19 cohort relative to the general cohort. Regrading *Outcome of delivery – finding*; for example, we observed a visit prevalence rate of 1.18% in the COVID-19 cohort, but only 0.0070% in the general cohort, yielding a prevalence ratio of 5.13. During normal times, deliveries only account for a small fraction of all inpatient visits. However, during the pandemic, there was a dramatic decrease in elective surgeries and hospitalizations besides those related to COVID-19 since hospital capacity was diverted their focus toward patients with COVID-19. In contrast, patients going into labor cannot be rescheduled and were regularly tested for SARS-CoV-2, which increased their representation within the COVID-19 cohort.

Potential associations between specific concepts of interest can be found through co-occurrence analysis. For instance, Table 3 confirms that the concepts associated with SARS-CoV-2 tests (ie, *Disease due to Coronavirus*, *Radiologic examination, chest; single view*, and *Infectious agent detection by nucleic acid [DNA or RNA]; severe acute respiratory syndrome coronavirus 2*) showed high co-occurrence prevalence with *Acute lower respiratory tract infection*, which shows natural strong associations between testing concepts for COVID-19 and one of the most prevalent concepts in the COVID-19 cohort.

Symptom prevalence analysis can be used to examine symptom-level characteristics of the cohorts. The COVID-19 cohort showed higher prevalence in dyspnea, fever, and cough symptoms than the other 2 comparator cohorts, which is concurrent with the known characteristics and symptoms of COVID-19 [20]. In contrast, a few of the known COVID-19 symptoms (eg, myalgia, throat pain, and chills) did now show high prevalence in the COVID-19 cohort, which might indicate that those symptoms are not clinically distinctively prevalent in patients with COVID-19.

Most of the results from the analyses align with those of existing studies, thus empirically validating the utility of COHD-COVID. COHD-COVID also can be used to find novel hypotheses related COVID-19. COHD-COVID can be used as cross-institutional data to validate or support other COVID-19 studies. For instance, the high prevalence ratio of *Cerebral infarction* in the COVID-19 cohort compared to that in the influenza cohort (Table 2) corroborates with reports from a few studies that SARS-CoV-2 might be more likely to cause thrombotic vascular events, including stroke, than other coronavirus and seasonal infectious diseases [21,22].

While we admit that the aggregated concept-level analyses may not be suitable to answer some clinical research questions, COHD-COVID will be useful for hypothesis generation and for validating emerging newly published findings on COVID-19 using real-world data. COHD, the precedent study of COHD-COVID, has been integrated into the National Center for Advancing Translational Sciences Biomedical Data Translator program, where the EHR-based data associations from COHD are linked to other sources of knowledge via knowledge graphs, allowing automated algorithms to perform reasoning on these knowledge graphs to answer biomedical questions and suggest novel hypotheses [23,24].

### Limitations

There are several limitations to this study, which should be noted. One of the limitations is that analyses performed in this study can be affected by the factors included in the data acquisition process (eg, change in the EHR system, human biases, and errors during entry). For example, coding trends and patterns (ie, the trend and pattern of frequently used concepts) can be changed through a shift in system. Another limitation is that multiple visits from the same patient can be used to calculate the metrics in the analyses since we used visit-based counts instead of patient-based counts for the analyses. This could affect the results of the specific concepts appearing in patients who are hospitalized more frequently. We also admit that some of results from the analyses cannot be validated by existing or up-to-date findings, considering rapidly growing and changing knowledge related to COVID-19. Thus, it is desirable to conduct a literature search before utilizing the data and results. We will update the data and results on a regular basis to further alleviate this limitation.

The EHR data used in this study were obtained from a single site: NYP/CUIMC. Even though NYP/CUIMC is a large academic medical center whose services cover the city and its surrounding areas, we admit that performing the analyses on the basis of the EHR data across multiple institutions and nations will be beneficial since multiple sites can diversify the population, improve accuracy, increase power and sensitivity to rare conditions, validate results by comparing across sites, and reduce variance that might exist in specific locations. Since the OMOP CDM provides the fundamentals to perform the same analyses on clinical data across different sites, we hope to collaborate with future studies sharing clinical characteristics of patients with COVID-19 and to generate a larger, richer, and more robust database that can be leveraged in translational research on COVID-19.

### Conclusions

In this study, we present COHD-COVID, a publicly accessible database providing useful clinical data for hospitalized patients with COVID-19, hospitalized patients with influenza, and general hospitalized patients. The analyses using the data from COHD-COVID confirmed the well-known clinical characteristics of patients with COVID-19 and can also be used to find novel hypotheses related to COVID-19. We expect COHD-COVID will provide researchers and clinicians quantitative measures of COVID-19–related clinical features to better understand and combat the pandemic.

## Abbreviations

API: application programming interface
COHD: Columbia Open Health Data
COHD-COVID: Columbia Open Health Data for COVID-19 Research
EHR: Electronic health records
NYP/CUIMC: NewYork-Presbyterian/Columbia University Irving Medical Center
OHDSI: Observational Health Data Science and Informatics
OMOP CDM: Observational Medical Outcomes Partnership Common Data Model
